# Human PI3Kγ deficiency and its microbiota-dependent mouse model reveal immunodeficiency and tissue immunopathology

**DOI:** 10.1038/s41467-019-12311-5

**Published:** 2019-09-25

**Authors:** Andrew J. Takeda, Timothy J. Maher, Yu Zhang, Stephen M. Lanahan, Molly L. Bucklin, Susan R. Compton, Paul M. Tyler, William A. Comrie, Makoto Matsuda, Kenneth N. Olivier, Stefania Pittaluga, Joshua J. McElwee, Debra A. Long Priel, Douglas B. Kuhns, Roger L. Williams, Peter J. Mustillo, Matthias P. Wymann, V. Koneti Rao, Carrie L. Lucas

**Affiliations:** 1 Department of Immunobiology, Yale University School of Medicine, New Haven, CT USA; 2 Clinical Genomics Program and Molecular Development of the Immune System Section, Laboratory of Immunology, NIAID, NIH, Bethesda, MD USA; 3 Human Immunological Diseases Section, Laboratory of Clinical Immunology and Microbiology, NIAID, NIH, Bethesda, MD USA; 4 Department of Comparative Medicine, Yale University, New Haven, CT USA; 5 Laboratory of Molecular Biology, Medical Research Council, Cambridge, UK; 6 Pulmonary Branch, Division of Intramural Research, NHLBI, NIH, Bethesda, MD USA; 7 Laboratory of Pathology, Clinical Center, NCI, NIH, Bethesda, MD USA; 8 Merck Research Laboratories, Merck & Co, Boston, MA USA; 9 Neutrophil Monitoring Laboratory, Applied/Developmental Research Directorate, Leidos Biomedical Research, Inc., Frederick National Laboratory for Cancer Research, Frederick, MD USA; 10 Division of Infectious Diseases and Immunology, Nationwide Children’s Hospital, Columbus, OH USA; 11 University of Basel, Department of Biomedicine, Basel, Switzerland

**Keywords:** Medical genetics, Adaptive immunity, Inflammation, Signal transduction, Translational immunology

## Abstract

Phosphatidylinositol 3-kinase-gamma (PI3Kγ) is highly expressed in leukocytes and is an attractive drug target for immune modulation. Different experimental systems have led to conflicting conclusions regarding inflammatory and anti-inflammatory functions of PI3Kγ. Here, we report a human patient with bi-allelic, loss-of-function mutations in *PIK3CG* resulting in absence of the p110γ catalytic subunit of PI3Kγ. She has a history of childhood-onset antibody defects, cytopenias, and T lymphocytic pneumonitis and colitis, with reduced peripheral blood memory B, memory CD8+ T, and regulatory T cells and increased CXCR3+ tissue-homing CD4 T cells. PI3Kγ-deficient macrophages and monocytes produce elevated inflammatory IL-12 and IL-23 in a GSK3α/β-dependent manner upon TLR stimulation. *Pik3cg*-deficient mice recapitulate major features of human disease after exposure to natural microbiota through co-housing with pet-store mice. Together, our results emphasize the physiological importance of PI3Kγ in restraining inflammation and promoting appropriate adaptive immune responses in both humans and mice.

## Abstract (web summary)

Causally linking a mutation to clinical phenotypes in rare hereditary diseases is both challenging and illuminating. Here the authors identify PI3Kɣ mutations in a patient with immune dysregulation, and recapitulate the phenotypes in PI3Kɣ-deficient mice by exposing them to natural microbiota from pet-shop mice.

## Introduction

Monogenic human immune disorders provide valuable insight into fundamental genes, proteins, and pathways that regulate function of the immune system. These insights not only enable precise therapies for patients with these inherited diseases but also elucidate novel and unexpected basic science insights applicable to many immune processes and disease states. Defects in hundreds of genes have been identified as causal drivers of such monogenic immune disorders, also referred to as inborn errors of immunity^1,2^. They result in states of immunodeficiency (with susceptibility to infections and/or malignancies) or immune dysregulation (with lymphoproliferation, autoimmunity, autoinflammation, and/or allergy), with a growing number of conditions encompassing both of these categories^3^.

Phosphatidylinositol 3-kinases (PI3Ks) are a family of lipid kinases that phosphorylate the 3-hydroxyl of phosphoinositides. Class I PI3Ks phosphorylate PtdIns(4,5)P_2_ to produce PtdIns(3,4,5)P_3_, a versatile second messenger that potently activates diverse signaling cascades to coordinate the cellular response to growth factors, cytokines, chemokines, and antigen receptor stimulation, among other inputs. In line with the ubiquitous nature of PI3K signaling in eukaryotes, dysregulated activity of PI3Ks contributes to many human diseases including cancer, overgrowth syndromes, and diabetes^4^. Moreover, we and others have reported immunodeficiency caused by heterozygous, gain-of-function mutations in the leukocyte-restricted *PIK3CD* or ubiquitously expressed *PIK3R1* gene, each encoding a subunit of the PI3Kδ complex^5–14^. However, defects in other PI3K genes have not been reported in patients with monogenic immune disease.

PI3Kγ is enriched in leukocytes (though also present in endothelial cells and cardiac myocytes) and consists of a catalytic p110γ subunit complexed with either a p84 or p101 regulatory subunit^15^. PI3Kγ was initially reported in mice to facilitate signaling downstream of G protein-coupled receptors (GPCRs) with particular emphasis on chemokine receptor responses in myeloid cells, which express more p110γ than lymphoid cells^16–18^. This feature has made PI3Kγ an attractive target for inhibition of leukocyte (especially granulocyte) trafficking to inflamed tissues in a spectrum of autoimmune diseases^19^, asthma^20^, high-fat diet-induced adiposity and insulin resistance^21^, and allograft rejection^22^. Additionally, defects in T cell and antibody responses in PI3Kγ knockout (KO) mice have been observed in some immunization and infection models^17,18,23,24^. More recently, the described functions of PI3Kγ have extended beyond GPCRs to include signaling downstream of receptor tyrosine kinases and TLR/IL-1R^25^ and suppression of anti-tumor immunity^26,27^ by myeloid cells. Mechanistically, PI3Kγ has been found to be activated by TLR stimulation in macrophages in a manner dependent on the Rab8a small GTPase^28–30^, though the biochemical connection between PI3Kγ and regulation of TLR-induced inflammation has not been fully elucidated. Nonetheless, a small molecule inhibitor (IPI-549) of PI3Kγ is now being tested in Macrophage Reprogramming in Immuno-Oncology (MARIO) trials to boost tumor immunity in humans^31^.

We report a human patient with bi-allelic loss-of-function mutations in *PIK3CG* encoding the p110γ catalytic subunit of PI3Kγ. She presented in childhood with antibody defects (and associated complications of autoimmune cytopenias and infections), lymphadenopathy/splenomegaly, and pathological T cell infiltration of barrier tissues (nasopharynx, lung, and gut). We further recapitulate key features of disease in *Pik3cg* KO mice by modeling the human condition through natural exposure to normal microbiota. Overall, our data are consistent with a mechanistic model in which deficiency in PI3Kγ results in GSK3-dependent overproduction of IL-12 and IL-23 by myeloid cells, leading to increased T cell accumulation in tissues that, together with defective immunoglobulin production and reduced regulatory T cells, underlies disease pathophysiology. These findings identify a condition that, for simplicity, could be termed “Inactivated PI3K-gamma Syndrome” (IPGS), clarify the parallel roles of PI3Kγ in restraining inflammation and promoting adaptive immunity, and provide important insights for optimizing efficacy and anticipating side effects of clinical PI3Kγ inhibitors.

## Results

### Patient with humoral defects and lymphocytic tissue infiltration

**Fig. 1:**
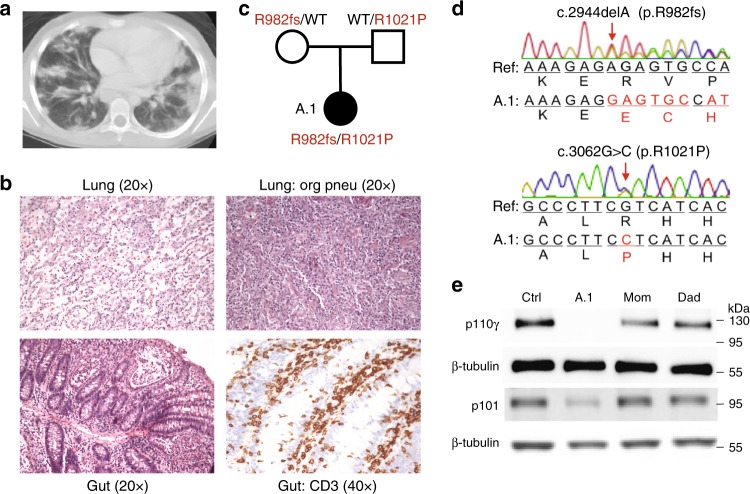
Patient with loss of PI3Kγ and T lymphocytic infiltration of lung and gut. **a** Chest computed tomography scan with diffuse pulmonary nodular and patchy infiltrates. **b** Hematoxylin and eosin staining of histological sections of a lung biopsy with lymphocytic infiltrates (top left) and regions of organizing pneumonia (top right) and gut biopsy with lymphocytic infiltrates (bottom left) and staining for CD3 (bottom right). **c** Pedigree with *PIK3CG* alleles inherited by patient A.1. **d** Chromatograms obtained by Sanger sequencing of *PIK3CG* genomic DNA from patient A.1. **e** Immunoblotting of p110γ, p101, and β-tubulin in T cell blasts from an unrelated healthy control, patient A.1, and parents

A female patient (hereafter called A.1) from a European-American family presented at nine years of age with fatigue and hemolytic anemia followed by early obstructive pulmonary impairment. A subsequent chest CT scan revealed bilateral nodular infiltrates and areas of patchy, peripheral-basal consolidation in lungs, and histological examination revealed a pattern of interstitial CD3+ lymphocytic infiltration, foamy histiocytes, scattered noncaseating granulomas, and luminal obstruction initially characterized as cryptogenic organizing pneumonia (Fig. 1a–b). Further follow up and analysis revealed clinical progression to hypogammaglobulinemia, thrombocytopenia, various lymphopenias, eosinophilia, mediastinal and hilar lymphadenopathy, and splenomegaly (Table 1). More recently, at sixteen years of age, patient A.1 developed enterocolitis with diarrhea and abdominal pain. Histological assessment of gut tissue revealed interstitial infiltrate of more than 25 CD3+ lymphocytes per 100 epithelial cells (Fig. 1b, bottom). Episodes of pneumonitis and colitis continue to recur intermittently, have an apparent noninfectious etiology (with separate incidences of infectious colitis), and respond to pulse doses of corticosteroids and steroid-sparing measures including mycophenolate mofetil.

**Table 1:** Clinical laboratory characteristics of patient A.1

	A.1	Reference range*
CD4:CD8 ratio	1.46–3.54	0.74–5.17
CD4+ T cells	427–795/μL 37.3–58.9%	358–1565/μL 27.0–62.2%
CD8+ T cells	132–342/μL ↓ 16.5–25.5% ↓	149–911/μL 11.2–50.8%
CD8+ central memory (CD62L+ CD45RA−)	14–38/μL ↓ 1.5–5.5%	25–180/μL 1.5–10.3%
CD8+ effector memory (CD62L− CD45RA−)	4–9/μL ↓ 0.4–1.3% ↓	24–175/μL 1.1–9.2%
CD8+ Temra (CD62− CD45RA+)	4–6/μL ↓ 0.6–0.6% ↓	11–172/μL 0.7–7.8%
CD4+ CD25+ T cells	114–224/μL ↓ 11.3–18.9% ↓	139–1223/μL 10.6–41.2%
DNαβT cells (CD4− CD8− TCRα/β+)	14–32/μL ↑ 1.3–3.0% ↑	2–23/μL 0.1–1.3%
γδT cells (TCRγ/δ+)	22–145/μL 2.2–11.5% ↑	5–261/μL 0.3–10.4%
CD4 + HLA-DR + T cells	20–115/μL ↑ 1.5–11.4% ↑	0–139/μL 0.0–6.8%
NK cells (CD56+ CD16+)	30–131/μL ↓ 2.9–12.9% ↓	87–729/μL 4.6–34.6%
Memory B cells** (CD20+ CD27+)	0–17/μL ↓ 0.0–1.5% ↓	12–118/μL 0.7–6.3%
IgG, serum***	294–413 ↓	549–1584 mg/dL
IgA, serum	15–33 ↓	45–359 mg/dL
IgM, serum	26–88 ↓	23–259 mg/dL
Neutrophils	1.18–6.96 K/μL ↓↑ 49.6–81.5% ↑	1.56–7.87 K/μL 29.8–77.0%
Eosinophils	0.043–0.570 K/μL ↑ 0.6–9.2% ↓↑	0.03–0.47 K/μL 0.0–7.0%
Monocytes	0.15–0.40 K/μL ↓ 3.5–10.5% ↓	0.19–0.86 K/μL 4.2–12.5%

A minimum of three tests from independent blood draws are included for each row. Percentages indicate % of lymphocytes for lymphocyte populations and % of leukocytes for non-lymphocyte populations.

*Listed ranges reflect the aggregate of ranges used by the NIH Clinical Center for each age (as established at the time of testing) at which blood draws from patient A.1 are included. In some instances, lab values fall outside the corresponding age-matched reference range but do not fall outside the aggregate range.

**Prior to receiving rituximab.

***Prior to receiving IVIG

The childhood of patient A.1 was remarkable for recurrent sinopulmonary, ear, skin, and urinary tract infections (commonly with *S. aureus*), chronic nasal congestion, and eczema. Additional episodes of colitis were sometimes associated with stool cultures positive for *C. difficile* and *Salmonella*. Vaccination responses were protective for tetanus, borderline protective for diphtheria, and protective for 4 of 23 pneumococcal strains. Warm autoimmune hemolytic anemia at nine years of age (preceding the initial pneumonitis by several months) was treated with steroids and blood transfusions; a recurrence of autoimmune cytopenias at seventeen years prompted CD20+ B cell depletion with rituximab. Patient A.1 is currently treated with immunoglobulin replacement therapy to restore humoral protection and mycophenolate mofetil to suppress inflammation.

### Compound heterozygous inheritance of deleterious *PIK3CG* alleles

Whole exome sequencing (WES) of genomic DNA from patient A.1 and her unaffected parents identified deleterious mutations in the *PIK3CG* gene encoding p110γ (Fig. 1c) and no other variants suspected of causing immune dysregulation. Patient A.1 inherited an allele from her healthy mother in whom a single base-pair deletion causes a frameshift beginning at R982 of p110γ, and an allele from her healthy father in whom a missense mutation results in an R1021P amino acid substitution in the kinase domain. The WES findings were confirmed by Sanger sequencing (Fig. 1d). The R1021P missense mutation is rare, affects a highly conserved residue, and is predicted to be damaging (CADD^32^ score of 34).

We assessed *PIK3CG* expression in T cell blasts and found reduced p110γ protein (using multiple detection antibodies) in both parents and virtually absent expression in patient A.1 compared to unrelated healthy control subjects (Fig. 1e). Abundance of the p101 regulatory subunit, which is unstable in the absence of p110γ, was also reduced in patient cells (Fig. 1e). The maternal allele is predicted to cause nonsense-mediated decay due to a premature stop codon in the penultimate exon, and instability of mRNA from this allele was supported by its low abundance (Supplementary Fig. 1A). The paternal R1021P substitution in the kinase domain destabilizes hydrogen bonds predicted to be necessary for protein stability (Supplementary Fig. 1B) and nearly completely abolishes in vitro recombinant kinase activity of p110γ alone or bound to either p84 or p101 subunit (Supplementary Fig. 1C). None of the other class I PI3K catalytic subunits (p110α, p110β, p110δ) nor the p85α regulatory subunit exhibited changes in protein expression in patient A.1 cells (Supplementary Fig. 1D).

Based on prior studies of PI3Kγ, we expected defective responses of patient A.1 neutrophils to GPCR ligands. However, under the conditions tested, we could not discern any consistent defects in reactive oxygen species (ROS) production or chemotaxis (Supplementary Fig. 1E–G). We did, however, observe a defect in phosphorylation of AKT and S6 in patient T cells after stimulation with the chemokines SDF-1α (Supplementary Fig. 2A–C) or CCL21 (Supplementary Fig. 2D) and found that the response to SDF-1α could be rescued by overexpression of wild-type (WT) p110γ in T cells from patient A.1 (Supplementary Fig. 2B–C). Nonetheless, in vitro chemotactic responses of patient T cells were not defective unless p110β, which can also be activated by GPCRs, was inhibited (Supplementary Fig. 2E). These data suggest that defective responses to chemokine receptors and other GPCRs may not be primary drivers of immune disease in patient A.1 with loss of p110γ, possibly due to compensation by p110β^33^.

### Reduced T_reg_ cells with increased inflammatory T cells and serum proteins

**Fig. 2:**
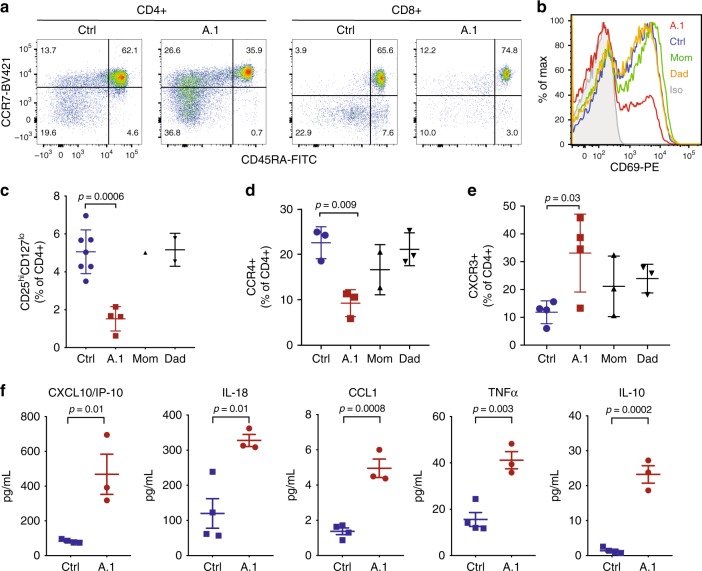
T cell abnormalities and elevated inflammatory serum cytokines/chemokines. **a** Flow cytometry of T cells from an unrelated healthy control or patient A.1 with staining for CD4, CD8, CD45RA, and CCR7, as indicated. **b** CD69 expression on CD8 + T cells from peripheral blood of the indicated subjects after 24 h of stimulation in vitro with anti-CD3 and anti-CD28 antibodies. **c** Frequency of CD25^hi^CD127^lo^ among CD4 + peripheral blood T cells in unrelated healthy controls (*n* = 7), patient A.1 (*n* = 4), mom (*n* = 1), and dad (*n* = 2). Data from four independent experiments is presented as mean ± SD. Statistical analysis was performed using one-way ANOVA with Dunnett’s multiple comparisons test. **d** Frequency of CCR4 + among CD4 + peripheral blood T cells in unrelated healthy controls (*n* = 3), patient A.1 (*n* = 3), mom (*n* = 2), and dad (*n* = 3). Data from four independent experiments are presented as mean ± SD. Statistical analysis was performed using one-way ANOVA with Dunnett’s multiple comparisons test. **e** Frequency of CXCR3 + among CD4 + peripheral blood T cells in unrelated healthy controls (*n* = 4), patient A.1 (*n* = 4), mom (*n* = 3), and dad (*n* = 3). Data from four independent experiments are presented as mean ± SD. Statistical analysis was performed using one-way ANOVA with Dunnett’s multiple comparisons test. **f** Concentrations of the indicated cytokine or chemokine in serum from independent blood draws of unrelated healthy controls (*n* = 4) and patient A.1 (*n* = 3). Data from three independent experiments are presented as mean ± SEM. Statistical analysis was performed using two-tailed unpaired T-test

To better understand the immunopathology in patient A.1, we further evaluated her peripheral blood lymphocytes. While frequencies of total B and CD4+ T cells in peripheral blood of A.1 (before rituximab administration) generally fell within age-matched normal ranges, frequencies of CD8+ T cells and CD20+ CD27+ memory B cells were consistently low (Table 1). Less consistent were derangements in frequencies and absolute counts of other circulating leukocyte populations, including increased double-negative (CD4-CD8-) T cells and eosinophils, reduced monocytes and platelets, and neutrophil counts falling either above or below clinically established normal ranges (Table 1). Among CD8 T cells, the large majority were naïve (CCR7+ CD45RA+ ), with reductions in the central memory (T_CM_; CCR7+ CD45RA−), effector memory (T_EM_; CCR7− CD45RA−), and terminally differentiated effector memory (T_EMRA_; CCR7− CD45RA +) compartments (Table 1 and Fig. 2a).

Functionally, patient T cells responded poorly to T cell receptor (TCR) stimulation in vitro, as determined by upregulation of surface activation marker CD69 (Fig. 2b), consistent with data in *Pik3cg* KO mice^17^. Inactivation of PI3Kγ through siRNA knockdown or p110γ inhibition was sufficient to recapitulate this activation defect, which could be overcome with very strong stimulation (Supplementary Fig. 2F–G). We next examined the frequency of regulatory T cells (T_reg_) in peripheral blood samples and observed a remarkably low frequency of CD25^hi^CD127^lo^ CD4+ T cells (Fig. 2c and Supplementary Fig. 2H) and FOXP3+ CD4+ T cells (Supplementary Fig. 2H–J) in patient A.1. Additionally, the expression level of FOXP3 is lower in patient T_reg_ cells (Supplementary Fig. 2I). Consistently, her CD4+ T cells also less frequently expressed the chemokine receptor and Th2/T_reg_ marker CCR4 (Fig. 2d). Moreover, expression of the chemokine receptor and Th1 marker CXCR3, which is involved in homing to inflamed tissues, was higher among CD4+ T cells (Fig. 2e), suggesting a bias toward Th1-type inflammatory responses. In support of this possibility, concentrations of the IFN-inducible CXCR3 ligand CXCL10 were elevated in the serum of patient A.1 (Fig. 2f). Additionally, the inflammatory IL-18, CCL1, and TNFα cytokines and anti-inflammatory IL-10 were significantly increased (Fig. 2f), while other cytokines were unchanged (Supplementary Fig. 2K).

Thus, PI3Kγ deficiency results in reduced T_reg_ cells and increased CXCR3+ tissue-homing Th1-like T cells, which could contribute to aberrant T cell infiltration of barrier tissues. However, T cell-intrinsic activation defects were relatively minor and could be overcome by strong stimulation, suggesting that T cell-extrinsic effects may be predominantly responsible for driving immunopathology. The presence of elevated inflammatory cytokines in serum from patient A.1, together with published work on regulation of TLR signals by PI3Kγ, led us to investigate the potential contribution of myeloid cells to disease.

### Myeloid cells overproduce IL-12 and IL-23 in a GSK3-dependent manner

**Fig. 3:**
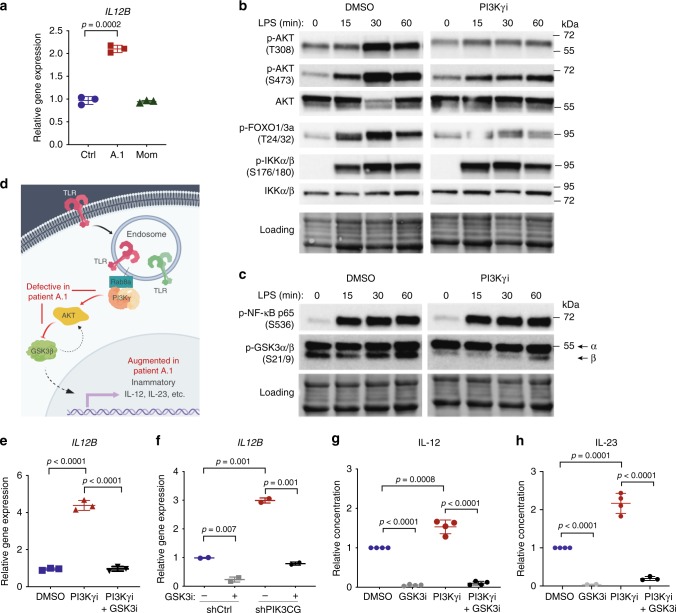
Macrophages/monocytes overproduce inflammatory IL-12 and IL-23 in a GSK3α/β-dependent manner upon TLR stimulation. **a** mRNA expression of *IL12B* normalized to *GAPDH* in monocyte-derived macrophages stimulated with IFNγ (20 ng/mL) and LPS (100 ng/mL) for 24 hr. Data are presented as mean ± SD, and are representative of two independent experiments in which one primary cell isolate for each group was run with three technical replicates. Statistical analysis was performed using a two-tailed unpaired T-test. **b**–**c** Immunoblotting for the indicated proteins in lysates from THP-1 monocytes stimulated with LPS (100 ng/mL) and treated with the PI3Kγ inhibitor IPI-549 (1 μM) or DMSO control. Equal protein loading was assessed using stain-free imaging (Bio-Rad). **d** Schematic of signaling pathway dysregulated in the absence of PI3Kγ activity (created with BioRender.com). **e** mRNA expression of *IL12B* normalized to *GAPDH* in THP-1 macrophages treated with PI3Kγi IPI-549 (1 μM) and GSK3i LY2090314 (20 nM) or DMSO control, as indicated, and stimulated with IFNγ (20 ng/mL) and LPS (100 ng/mL) for 16 h. Data are presented as mean ± SD, and are representative of three independent experiments, in which one primary cell isolate for each group was run with three technical replicates. Statistical analysis was performed using one-way ANOVA with Tukey’s multiple comparisons test. **f** mRNA expression of *IL12B* normalized to *GAPDH* and *RPL37A* in THP-1 macrophages expressing *PIK3CG*-targeted or control shRNA treated as in **e** and stimulated for 8 hr with IFNγ (20 ng/mL) and LPS (100 ng/mL). Data are presented as mean ± CI, and are representative of two independent experiments in which one primary cell isolate for each group was run with two technical replicates. Statistical analysis was performed using two-tailed unpaired T-test. **g**–**h** Cytokine protein concentration (relative to DMSO condition) in supernatant of healthy human donor monocyte-derived macrophages (**g**) or monocytes (**h**) treated with IPI-549 (500 nM) and/or GSK3 inhibitor LY2090314 (20 nM), as indicated, and stimulated with LPS (200 ng/mL) and IFNγ (5–35 ng/mL). Data are presented as mean ± SD and combined from 3–4 independent experiments in which one primary cell isolate for each group was run in duplicates. Statistical analysis was performed using two-tailed unpaired T-test

Pathological tissue inflammation can be initiated by responses of innate immune cells to microbial products that bind pattern recognition receptors (e.g., TLRs), leading us to hypothesize that T cell infiltration in tissues of patient A.1 could be at least partly driven in a cell-extrinsic manner by hyperinflammatory innate cells. Consistent with published reports^26–30^, we found that patient monocyte-derived macrophages stimulated with LPS (TLR4 agonist) and IFNγ exhibited elevated expression of the pro-inflammatory cytokine gene *IL12B* (Fig. 3a), as well as *IL1B* and *TNF* (Supplementary Fig. 3A–B). We next assessed signaling responses to LPS to elucidate the relevant signals downstream of PI3Kγ. In human THP-1 macrophages treated with the IPI-549 PI3Kγ inhibitor^34^, we observed a reduction in LPS-induced phosphorylation of AKT (T308 and S473), FOXO1/3a (T24/32), and GSK3β (S9) but no effect on phosphorylation of IKKα/β (S176/180) or NF-κB p65 (S536) (Fig. 3b–c and Supplementary Fig. 3E). Since glycogen synthase kinase (GSK) 3β has been implicated in regulation of inflammatory responses by macrophages^35^, we hypothesized that PI3Kγ deficiency results in unrestrained pro-inflammatory signals from hyperactive GSK3β due to reduction in inhibitory S9 phosphorylation (Fig. 3d). Indeed, LPS/IFNγ-induced *IL12B* (and *IL1B*) expression in THP1 cells was augmented by inhibition or knockdown of PI3Kγ, and this effect was reversible by inhibiting GSK3α/β using nanomolar concentrations of LY2090314^36^ (Fig. 3e–f, Supplementary Fig. 3C–D, F–G). We recapitulated this observation in primary human monocytes, assessing secretion of the two pro-inflammatory cytokine proteins that share the subunit encoded by *IL12B*, IL-12 and IL-23 (Fig. 3g–h). A feedback loop between GSK3 and AKT has been described based on the observation that knockdown of GSK3 reduces AKT phosphorylation^37^. Since PI3Kγ deficiency itself results in reduced AKT phosphorylation in myeloid cells, rescue of the inflammatory phenotype by GSK3 inhibition in this context is unlikely to be related to further reduction in AKT phosphorylation. Indeed, we found no evidence for reduced AKT or S6 phosphorylation in THP1 cells under conditions of GSK3 inhibition (Supplementary Fig. 3H). Moreover, NF-κB p65 phosphorylation on S536 was unaffected despite robust stabilization of β-catenin by GSK3 inhibition (Supplementary Fig. 3I–J).

Thus, human PI3Kγ deficiency reduces AKT activation and promotes a pro-inflammatory macrophage phenotype with markedly increased IL-12 and IL-23 production in a GSK3-dependent manner.

### Recapitulation in *Pik3cg* KO mice after exposure to natural microbiota

**Fig. 4:**
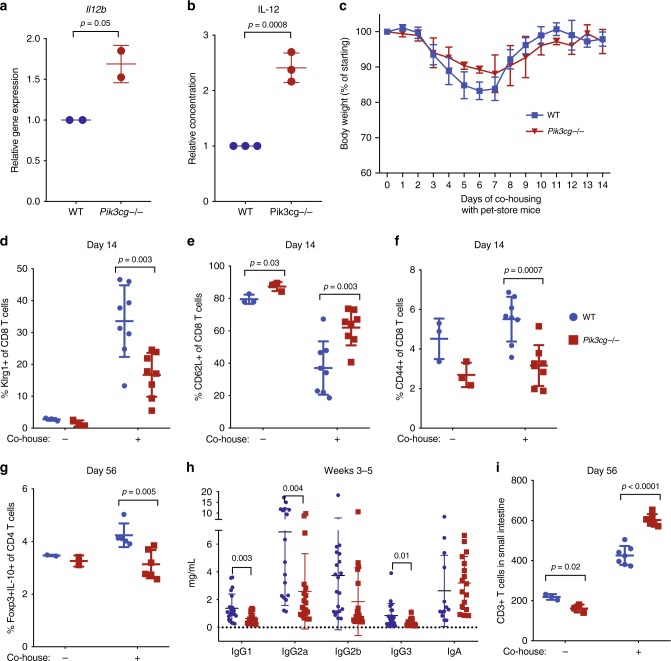
*Pik3cg*-deficient mice recapitulate major features of human disease after exposure to natural microbiota through co-housing with pet-store mice. **a** mRNA expression of *IL12b* normalized to *Hprt1* and *Rpl13a* in murine bone marrow-derived macrophages stimulated with LPS (100 ng/mL) and IFNγ (5 ng/mL) for 24 h. Data from two independent experiments (each with n = 2/group) are presented as mean ± SD. **b** Supernatant IL-12 concentration from murine bone marrow-derived macrophages stimulated with LPS (100 ng/mL) and IFNγ (5 or 20 ng/mL). Data are presented as mean ± SD and combined from three independent experiments. **c** Body weight of *Pik3cg*−/− and wild-type mice co-housed with pet-store mice. Data are representative of four independent experiments. **d**–**f** Frequency of Klrg1+ , CD62L+ , and CD44+ among CD8+ T cells from animals with or without exposure to pet-store mice at day 14. Data are presented as mean ± SD and are representative of four independent experiments (n = 3 for each non-co-housed group and n = 8 for each co-housed group). **g** Frequency of IL-10+ Foxp3+ cells among CD4 + T cells after PMA/ionomycin stimulation of splenocytes from WT (n = 2 for non-co-housed and n = 5 for co-housed) and *Pik3cg*−/− (n = 2 for non-co-housed and n = 6 for co-housed) after 56 days. Data are presented as mean ± SD. **h** Concentration of antibodies in serum from animals at 3–5 weeks after exposure to pet-store mice. Data are presented as mean ± SD, summarized from four independent experiments (n = 17–21 for each group). Statistical analysis was performed using unpaired T-test validated with Holm-Sidak method. **i** Total number of CD3 + T cells by immunohistochemistry per 200 μm section of small intestine from animals with or without exposure to pet-store mice at day 56. Data are presented as mean ± SD and representative of three independent experiments (n = 3 for non-co-housed and n = 7 for co-housed WT; n = 3 for non-co-housed and n = 6 for co-housed *Pik3cg*−/−). Statistical analysis was performed using two-tailed unpaired T-test in **a**–**g** and **i**

With only one patient identified so far with bi-allelic *PIK3CG* mutations, we sought to independently validate physiologically relevant consequences of PI3Kγ deficiency in vivo that were not necessarily expected from prior work (i.e., global immunoglobulin defects, T_reg_ cell defects, and T cell infiltration of lung/gut). We first confirmed that bone marrow-derived macrophages (BMDM) from *Pik3cg* KO mice (Supplementary Fig. 4A) produced more IL-12 in vitro (Fig. 4a–b) and had normal kinetics of NF-κB activation (Supplementary Fig. 4B). We next sought to replicate the human condition by exposing co-housed WT and *Pik3cg* KO mice to natural pathogens/microbiota through addition of healthy pet-store mice to their cages, which has been reported to alter immune traits in laboratory mice to more closely mirror those in humans^38,39^. Upon co-housing with pet-store mice, WT and KO animals exhibited similar weight loss and survival (Fig. 4c and Supplementary Fig. 4C) over a 60-day period with no clinical signs of long-term illness. Additionally, both groups of mice exhibited increased serum TNFα and IFNγ and tested positive for pathogens present in the pet-store mice (Supplementary Fig. 4D, Supplementary Table 1). However, upregulation of Klrg1 and CD44 with concomitant downregulation of CD62L on CD8 T cells assessed on Day 14 was significantly impaired in KO mice (Fig. 4d–f and Supplementary Fig. 4E–G), consistent with activation defects. In line with observations in patient A.1, we observed a significantly reduced T_reg_ frequency in *Pik3cg* KO animals after long-term exposure to pet-store mice (Fig. 4g–h and Supplementary Fig. 4H). Total serum immunoglobulin levels were assessed in WT and KO animals co-housed for 3–5 weeks with pet-store mice, and all isotypes tested trended lower in KO mice, with IgG1, IgG2a, and IgG3 significantly reduced (Fig. 4i and Supplementary Fig. 4I). Furthermore, T cell infiltration of the small intestine was significantly greater in KO mice (Fig. 4j), and a similar trend was observed in the lung but did not reach significance when cage swapping was used to expose laboratory mice to pet-store mouse pathogens (Supplementary Fig. 4J). We performed complete blood counts and did not observe signs of anemia (Supplementary Fig. 4K). Overall, our findings show that PI3Kγ normally functions in two likely interrelated processes: blunting inflammatory myeloid cell responses and promoting normal adaptive immune responses. The immunopathology and broadly defective humoral responses from PI3Kγ deficiency were not predicted from prior mouse studies; however, prompted by human data from patient A.1, these major phenotypes were elicited in *Pik3cg* KO mice upon natural exposure to physiologically relevant mouse pathogens.

Thus, we observe overlapping phenotypes in human and mouse PI3Kγ deficiency when normal environmental exposure to pathogens occurs, and the major disease features include reduced markers of T cell activation, low T_reg_ cell frequency, low immunoglobulin production, and increased T cell infiltration in the gut, associated with hyper-inflammatory macrophages.

## Discussion

We have identified the first patient with bi-allelic, deleterious mutations in *PIK3CG*, resulting in loss of the catalytic p110γ subunit of the leukocyte-enriched PI3Kγ complex. Effects of p110γ deficiency have been examined in mouse models, and our work now provides insight into physiological roles for human PI3Kγ that not only defines a monogenic human condition but also reveals fundamental biology relevant to design of immune-modulating therapies. Patient A.1 suffers from antibody defects (autoimmune cytopenias and low immunoglobulin, or common variable immunodeficiency (CVID), with recurrent sinopulmonary infections) and lung/gut disease characterized by pathological accumulation of T cells. This overall phenotype would not have been predicted from prior animal studies; however, many individual features bear resemblances to the phenotypes already reported in *Pik3cg*−/− mice. Namely, both mouse and human exhibit in vitro T cell activation defects, some form of in vivo antibody response abnormalities, and exaggerated pro-inflammatory cytokine production by myeloid cells in vitro^16–18,23,24,26,27,29^. Potentially contrasting findings from the literature include defects in neutrophil function observed in mice but not human (under the conditions tested) and inflammatory T cell infiltration of lung/gut, low T_reg_ cells, and autoimmune cytopenias occurring in human but not spontaneously observed in laboratory mice. Additionally, PI3Kγ deficiency or inhibition has previously been reported to prevent efficient induction of CXCR3 expression on activated mouse T cells^40^, in contrast to our finding of increased CXCR3 + T cells in patient A.1. We speculate that this discrepancy likely relates to T cell-intrinsic activation defects at early timepoints resulting in reduced CXCR3 upregulation described by Barbi, et al. and more complex, long-term stimulation of T cells in the context of high IL-12/IL-23 from myeloid cells at barrier tissues in patient A.1^40^.

We additionally hypothesize that defects in neutrophil responses were not observed in patient A.1 because they may have been masked by pre-priming in the context of in vivo inflammation^41^, p110β may compensate for p110γ in humans, or both. Indeed, we observed normal chemotactic responses to chemokines in patient cells unless p110β was inhibited, consistent with redundancy of p110β and p110γ in this function^33^. Thus, we pursued the hypothesis that loss of p110γ in humans causes disease, at least in part, from dysregulation of non-GPCR pathways.

Besides GPCR signaling and leukocyte chemotaxis, p110γ has recently been described to be activated by TLR stimulation in macrophages upon binding to Rab8a on macropinosomes, resulting in reduced inflammatory cytokine production^26–30^. Indeed, myeloid cells from patient A.1 (or healthy donor cells treated with the IPI-549 PI3Kγ inhibitor) exhibited increased IL-12 and IL-23 production, and we advanced molecular insights by demonstrating that this phenotype in PI3Kγ-deficient cells could be reversed by inhibiting GSK3, a kinase normally negatively regulated by PI3K signaling. GSK3 has been described to feed back on AKT such that loss of GSK3 reduces AKT phosphorylation; however, we see no evidence of this under the conditions we tested in myeloid cells with pharmacologic GSK3 inhibition^37^. IL-23 had not previously been reported to be elevated in PI3Kγ-deficient myeloid cells; however, it is implicated in inflammation and may be relevant for disease. Thus, we envision a model in which TLR-expressing cells residing in barrier tissues of patient A.1 are hyper-responsive to microbial products, and this imbalance drives an inflammatory process with elevated CXCL10 production and aberrant recruitment of T cells expressing the CXCR3 receptor (Supplementary Fig. 6). High IL-10 in serum from patient A.1 may reflect increased production in response to high local IL-12^42,43^.

Lack of a spontaneous inflammatory lung/gut phenotype in *Pik3cg*-deficient mice may be partially reconciled by the disparate environmental exposures in mice and humans. Indeed, specific pathogen-free environments for laboratory mice are known to affect immune system development and may have a particularly significant impact at barrier tissues, including lung and gut^38^. Our studies of the effects of natural pathogen exposure in *Pik3cg*-deficient mice co-housed with pet-store mice support this hypothesis and further underscore the utility of this approach. We observed that ‘dirty’ *Pik3cg* KO mice shared many of the features of disease in patient A.1. Specifically, both exhibited elevated *IL12B* from monocytes/macrophages, reduced T cell activation, defective immunoglobulin production, increased T cell infiltration in gut tissue, and a reduced frequency of T_reg_ cells. Unlike patient A.1, *Pik3cg* KO mice did not exhibit higher than WT levels of inflammatory cytokines/chemokines in the serum or overt clinical signs of disease, which may be due to the necessarily short mouse experiments relative to the years of disease development experienced by patient A.1. Additionally, under the conditions tested with cage swapping as a relevant variable for respiratory pathogen transmission, the increase in T cell infiltration of the lung tissue was not significant and skewing toward CXCR3+ T cells was not observed. Anemia is another feature of human disease not observed in the ‘dirty’ KO mice. Although the pet-store mouse exposure method inherently lacks the consistency and simplicity of controlled single-infection experiments, its ability to model the broad exposures and unknowns encountered by humans, especially those with primary immunodeficiencies, makes it translationally relevant for study of immune responses and function. Future efforts in the mouse model will expand our understanding of PI3Kγ and disease resulting from its absence through investigation of the key cell types and cytokines driving disease in ‘dirty’ and singly-infected *Pik3cg*-deficient mice.

One of the surprising features of disease in p110γ-deficient patient A.1 is the marked reduction in frequency of T_reg_ cells, considering evidence that T_reg_ cells actively downregulate overall PI3K activity to maintain suppressor function and FOXP3 expression^44–46^. In fact, PI3Kγ inhibition has been reported to boost T_reg_ cell function and ameliorate disease severity in mouse models of colitis^47^ and autoimmune diabetes^48^. We hypothesize that in vivo TLR stimulation in the context of PI3Kγ-deficiency leads to augmented Th1-driving cytokines such as IL-12 from myeloid cells that could destabilize T_reg_ cell identity and confer a Th1-like effector phenotype, as previously described^49,50^. Additionally, the TCR-induced activation defect in T cells may reduce production of IL-2 required to sustain T_reg_ cells^51^ or cause defects in thymic T_reg_ cell selection^52,53^.

Overall, T_reg_ cell defects may contribute to autoantibody-driven features of disease in patient A.1 (i.e., autoimmune cytopenias), as has been shown in human T_reg_ deficiency^54^. Interestingly, the disease phenotype observed in patient A.1 is reminiscent of other primary immune disorders with T_reg_ defects. For example, patients with loss-of-function mutations affecting CTLA-4^55^ or its intracellular trafficking partner LRBA^56^ exhibit T_reg_ dysfunction manifesting in multi-organ (including lung and gut) autoimmune infiltration. Defects in humoral immunity are also common features of these disorders, and excessive Th1 responses have been hypothesized to cause the observed hypogammaglobulinemia and reduced frequency of class-switched memory B cells^55^ in addition to the generation of autoantibodies to erythrocytes, thrombocytes, and neutrophils^57^.

In conclusion, we have described a monogenic human condition and mouse model for ‘Inactivated PI3K-gamma Syndrome’ (IPGS). Human IPGS is associated with immunodeficiency and immune dysregulation, including antibody defects and inflammatory lung/gut disease with lymphocytic infiltration. This disease can be modeled by natural pathogen exposure in PI3Kγ-deficient mice, enabling future testing of tailored therapies such as blocking anti-IL-12/23 antibody (e.g., ustekinumab) or GSK3 inhibitors in IPGS and related disorders. Our findings further underscore the critical nature of PI3Kγ in immune system regulation and raise awareness of possible side effects that can be expected upon long-term pharmacological targeting of this enzyme to treat human disease, as in the MARIO cancer trials. In particular, patients receiving PI3Kγ inhibitors should be monitored for antibody defects and lymphocytic infiltration at barrier tissues, particularly when combined with other immunotherapies such as immune checkpoint blockade.

## Methods

### Human subjects

All human subjects in this study provided written informed consent to use their samples for research and to publish de-identified genetic sequencing data, in accordance with Helsinki principles for enrollment in research protocols that were approved by the Institutional Review Boards of the National Institute of Allergy and Infectious Diseases, National Institutes of Health (NIH) and of Yale University. Blood from healthy donors was obtained at the NIH Clinical Center and at Yale under approved protocols. All relevant ethical regulations for work with human participants were followed.

### Histology

Hematoxylin & Eosin (H&E) stained sections from right upper lobe, mediastinal lymph node (from 2008) and multiple gastrointestinal biopsies (upper and lower endoscopies) from 2015, 2016 and 2017 were reviewed. Immunohistochemical stains were performed on formalin fixed paraffin embedded tissue sections using antibodies against CD20, CD3, CD45RO, CD4, CD8, and S100. The stains were performed on an automated system (BenchMark XT and BenchMark Ultra) according to the manufacturer’s instructions. Images were taken with an Olympus Bx41 microscope, objective UPLanFI 20×/0.50 ∞/0.17 and 40×/0.75∞/0.17 with an adaptor U-TV0.5xC using a digital camera Q-imaging Micropublisher 5.0 RTV. The images were captured using “Q-capture Version 3.1” and imported into Adobe Photoshop CC 2017.

### DNA sequencing

Genomic DNA was isolated from PBMCs from patient A.1 and parents. SureSelect Human All Exon 50 Mb kit (Agilent Technologies) coupled with massively parallel sequencing by Illumina HiSeq Sequencing System was performed using the collected DNA. For individual samples, WES produced ∼50–100x sequence coverage for targeted regions. For confirmation of mutations in the patients, genomic DNA was PCR amplified and Sanger sequencing of purified PCR amplified products was performed.

### Whole-exome sequence analysis

All sequenced DNA reads were mapped to the hg19 human genome reference by Burrows-Wheeler Aligner with default parameters. Single nucleotide variant and indel calling were performed using the Genome Analysis Toolkit (the Broad Institute). All SNVs/indels were annotated by SeattleSeq Annotation and an in-house custom analysis pipeline was used to filter and prioritize for autosomal recessive or de novo disease-causal variants based on the clinical pedigree for patient A.1.

### Cell culture

Human PBMCs were isolated by Ficoll-Paque PLUS (GE Healthcare) or Lymphoprep (STEMCELL Technologies) density gradient centrifugation, washed twice in PBS, and resuspended at 10^6^ cells/ml in complete RPMI 1640 (cRPMI) medium (Lonza) containing 10% FBS, 2 mM glutamine, and 100 U/mL each of penicillin and streptomycin (Invitrogen). Cells were activated with 1 µg/mL anti-CD3 (clone HIT3α; BD) and 1 µg/mL anti-CD28 (clone CD28.2; BD). After 3 days, activated T cells were washed and then cultured in complete RPMI-1640 medium supplemented with 100 U/mL recombinant human IL-2 (rhIL-2; R&D Systems). Cells were also activated with Dynabeads Human T-activator CD3/CD28 (ThermoFischer Scientific) with a bead-to-cell ratio of 1:1 for 24 h. Cells were separated using EasySep Human T Cell Isolation Kit (STEMCELL Technologies), and isolated T cells were activated with 1.25 or 2.5 μM of anti-CD3 (clone HIT3α; BD) and 2 μg/mL anti-CD28 (clone CD28.2; BD) for 24 h. To generate human monocyte-derived macrophages, CD14+ CD16− monocytes were isolated from PBMCs using the EasySep Human Monocyte Isolation Kit (STEMCELL) and cultured for 7 days in cRPMI with 50 ng/mL recombinant human m-CSF (Biolegend) and 25 ng/mL of recombinant human IL-10 changing media every 2 days^58^. Macrophages or undifferentiated monocytes were treated with 0.5–1 μM PI3Kγ inhibitor IPI-549 (MedChem Express), 20 nM GSK3 inhibitor LY2090314 (MedChem Express) or DMSO (Sigma-Aldrich) control and stimulated for 24 h with 100 ng/mL LPS (Sigma, L2654) and 5–35 ng/mL recombinant human IFNγ (Peprotech) before extracting RNA or using cell-culture supernatants to quantify cytokine concentrations. THP-1 monocytes were differentiated to a macrophage-like state by stimulation with PMA (100 or 300 nM) for 48 hr followed by 24-hr culture in cRPMI without PMA. THP-1 macrophages were treated with 0.5–1 μM PI3Kγ inhibitor IPI-549 (MedChem Express), 20 nM GSK3 inhibitor LY2090314 (MedChem Express) or DMSO (Sigma-Aldrich) control and stimulated with 100 ng/mL LPS (Sigma, L2654) and 20 ng/mL recombinant human IFNγ (Peprotech) before extracting RNA. To produce mouse bone marrow-derived macrophages, mice were sacrificed and tibias and femurs were flushed with incomplete DMEM. Cells were washed and then plated in complete DMEM (cDMEM) supplemented with 20 ng/mL m-CSF for 7 days (media + MCSF). Mouse bone marrow derived macrophages were stimulated with 100 ng/mL LPS (Sigma, L2654) and 5 ng/mL IFNγ (Peprotech) for 24-hr in cDMEM before extracting RNA or using cell-culture supernatants to quantify cytokine concentrations.

### Flow cytometry on human samples

For standard surface staining, PBMCs (10^6^ cells/sample) were washed with PBS and incubated for 30 min at 4 °C (dark) in 100 µl PBS plus 2% FBS with indicated fluorochrome-labeled monoclonal antibodies. After washing with PBS two times, 30,000–50,000 live cells were analyzed by flow cytometry. For phospho-flow staining, cells were kept in RPMI while alive, fixed directly in RPMI using Lyse/Fix (BD), and then permeabilized with Perm Buffer III (BD) according to the manufacturer’s instructions and stained with the indicated antibody or isotype control. For transcription factor staining, cells were fixed and stained with True-Nuclear™ Transcription Factor Buffer Set (Biolegend) according to the manufacturer’s instructions and stained with the indicated antibody or isotype control. For surface staining, anti-CD3 PE/Cy7 (SK7), anti-CD3 PerCP/Cy5.5 (UCHT1), anti-CD4 APC/Cy7 (RPA-T4), anti-CD8 BV650 (RPA-T8), anti-CD25 PE/Cy7 (BC96), anti-CD45RA FITC (HI100), anti-CD69 PE (FN50), anti-CCR4 PerCP/Cy5.5 (L291H4), anti-CCR7 BV421 (G043H7), anti-CD127 AF488 (A019D5), and anti-CXCR3 Alexa Fluor 647 (G025H7) were used, all from BioLegend. Additionally, anti-CD25 BV421 (M-A251) from BD Biosciences was used. For phospho-flow, anti-pAKT Ser473 Alexa Fluor 647 (D9E) from Cell Signaling Technology was used. For transcription factor staining, anti-FOXP3 Alexa Fluor 488 (150D) from BioLegend, and anti-FOXP3 PE (259D/C7) from BD Biosciences was used. Gating strategies are included as Supplementary Fig. 5.

### Immunoblotting

Cells were washed in ice-cold PBS without serum and lysed on ice in 1% Triton X-100, 50 mM Tris-Cl, pH 8, 150 mM NaCl, 2 mM EDTA, 10% glycerol, complete protease inhibitor cocktail (Roche), and phosphatase inhibitor cocktails (Sigma-Aldrich). The lysates were then clarified by centrifugation at 15,000 *g* at 4 °C for 10 min, 2x sample buffer (Quality Biological) completed with 5% beta-mercaptoethanol was added, and lysates were boiled at 95 °C for 5 min. Approximately 20 µg of total protein was separated using stain-free SDS-PAGE gels and transferred to a nitrocellulose membrane (Bio-Rad). Images of total protein loading using Bio-Rad stain-free imaging were obtained, and membranes were blocked with 5% nonfat dry milk in Tris-buffered saline (TBS) with 0.01% Tween-20 (TBST) for 1 h at room temperature before incubating with primary antibody overnight at 4 °C. After washing with TBST for 1 h at room temperature, HRP-conjugated secondary antibody was added for an additional hour at room temperature. After a final 1-h wash step, HRP substrate (Clarity, Bio-Rad) was added to the membranes, which were then subjected to chemiluminescent imaging using the ChemiDoc Touch Imaging System. The following validated primary antibodies purchased from Cell Signaling Technology: anti-p110γ (4252), anti-p110α (C73F8), anti-p110β (C33D4), anti-p110δ (D1Q74), anti-p85α (6G10), anti-p101 (D32A5), and anti-β-tubulin (9F3), anti-p-AKT T308 (C31E5E), anti-p-AKT S473 (D9E), anti-AKT (pan) (C67E7), anti-p-FOXO1/3a T24/32 (9464), anti-p-IKKα/β Ser176/180 (16A6), anti-IKKα/β (2682), anti-p-NF-κB p65 S536 (93H1), and anti-p-GSK-3α/β (D17D2). Secondary antibodies were from SouthernBiotech, and an additional anti-p110γ antibody was generously provided by Dr. Emilio Hirsch. We provide an uncropped image of a p110γ western blot in cells from patient A.1 compared to controls in Supplementary Fig. 1H.

### Protein expression and purification of PI3Kγ

Expression of wild-type and R1021P mutant of p110γ-His6 either alone or in a complex with p84 or p101 regulatory subunit was conducted in Sf9 insect cells. The p110γ/p84 and p110γ/p101 complexes were co-expressed from a single plasmid using a MultiBac expression system. The genes were assembled into a single plasmid using a “multiplication module” approach where a I-CeuI/BstXI fragment obtained by digestion of a p110γ (wild-type or R1021P mutant)/pAceBac1 plasmid was ligated into a I-CeuI–digested p84 or p101/pAceBac1 vector. Expression was done in Sf9 insect cells. Cells were infected at density of 2.0 × 10^6^ cells/mL and harvested after 55 h of infection, lysed by sonication for 4 min and centrifuged for 30 min at 140,000 g. The p110γ alone and p110γ/p84 were purified by a 4-step purification procedure, consisting of a 5 mL HisTrap FF, 5 mL HiTrap Q HP, 5 mL HiTrap Heparin HP and Superdex 200 16/60 gel filtration column, while p110γ/p101 was purified with a 3-step purification protocol consisting of HisTrap FF, HiTrap Q HP and Superdex 200 16/60. The gel filtration column was equilibrated with 20 mM Tris, pH 7.5, 100 mM NaCl, 1 mM (NH_4_)_2_SO_4_, and 2 mM DTT (for wild-type) or 20 mM Tris, pH 7.5, 100 mM NaCl, 1 mM (NH_4_)_2_SO_4_, 5% (v/v) glycerol and 2 mM DTT (for R1021P mutant). The proteins were concentrated to 2–3 mg/mL, frozen in liquid nitrogen, and stored at −80 °C.

### Lipid kinase assays

Purified PI3Kγ proteins were assayed for PtdIns(3,4,5)P_3_ formation using competitive fluorescence polarization assay (Echelon Biosciences, Salt Lake City, UT, USA) carried out in 384-well microtitre plates. Lipid vesicles (sonicated and extruded through a 100 nm filter) were used at a final concentration of 1 mg/mL and contained 5% (w/w) sphingomyelin/10 % (w/w) cholesterol/ 15% (w/w) porcine brain phosphatidylcholine/45% (w/w) porcine brain phosphatidylethanolamine/20% (w/w) porcine brain phosphatidylserine/ 5% porcine brain PtdIns(4,5)P_2_. Lipid vesicles were mixed with each PI3Kγ construct, in a final buffer containing 20 mM HEPES pH 7.5, 150 mM NaCl, 1 mM tris(2-chloroethyl)phosphate (TCEP), 6 mM MgCl_2_ and 0.3 mM ATP in a total volume of 10 μL. Reaction was carried out for 30 min at room temperature, and quenched by adding 5 μL of stop solution contained 20 mM HEPES pH7.5, 150 mM NaCl, 1 mM TCEP, 30 mM EDTA and 400 nM GST-Grp1-PH, followed by adding 5 μL of 2.5 μM TAMRA-Ins(1,3,4,5)P_4_ in HNT buffer (20 mM HEPES pH 7.5, 150 mM NaCl, 1 mM TCEP). The plate was read in a PHERAstar spectrofluorometer (BMG Labtech, Ortenberg, Germany) with the FP/540–20/590–20/590–20 optical module. Standard curves were performed with diC8-PtdIns(3,4,5)P_3_ in the presence of lipid vesicle.

### *PIK3CG* shRNA knockdown

shRNA oligos targeting human *PIK3CG* (TRCN0000199330) were cloned into the pLKO.1 vector (Addgene plasmid #10878). 293 T cells were transfected with the constructed shRNA-containing pLKO.1 transfer plasmid or scramble shRNA negative control (Addgene plasmid #1864), psPAX2, and pCMV-VSV-G to generate shRNA lentiviruses and harvest them from supernatants^59^. Stable gene knockdown cell lines were generated by lentiviral transduction of THP-1 cells, followed by puromycin selection.

### Serum and supernatant protein analysis

Human serum concentrations of CXCL9, CXCL10, and CXCL11 were measured using the LEGENDplex Human Proinflammatory Chemokine Panel (BioLegend) according to the manufacturer’s instructions. Human serum concentrations of other cytokines were measured using the 42-Plex Human Cytokine/Chemokine Array (Eve Technologies). Mouse serum antibody concentrations were measured using the LEGENDplex Mouse Immunoglobulin Isotyping Panel (BioLegend). Mouse serum cytokine and chemokine concentrations were measured using the LEGENDplex Mouse Inflammation Panel (BioLegend). Cytokine concentrations from cell culture supernatant were measured with the Mouse IL-12 (p70) ELISA MAX Deluxe, Human IL-12 (p70) ELISA MAX Deluxe, and LEGEND MAX Human IL-23 ELISA Kit (all from BioLegend).

### Neutrophil functional assays

ROS production assays and chemotaxis assays were performed as previously described^60^. Briefly, human polymorphonuclear neutrophils (PMNs) from blood were isolated using Ficoll-Paque density gradient, dextran sedimentation, hypotonic lysis (to remove remaining red blood cells), and washed. Quantitative analysis of extracellular ${\mathrm{O}}_2^{\bar \cdot }$ production was performed by superoxide dismutase-inhibitable ferricytochrome c reduction. PMNs (0.25 × 10^6^/mL HBSS HEPES, pH 7.4) were incubated at 37 °C with cytochrome c (100 μM) and catalase (100 μg/mL) for 15 m after the addition of either buffer, PMA (100 ng/mL) or fMLP (10^−4−10−7^ M); or for 30 m after the addition of opsonized zymosan (1.0 mg/mL). The reaction tubes were spun, and the supernatant fluid analyzed spectrophotometrically for ${\mathrm{O}}_2^{\bar \cdot }$–dependent reduction of cytochrome c using an analytical wavelength of 549.5 nm. Superoxide dismutase (10 μg/mL) was added to an identically prepared reaction tube to serve as a blank. ${\mathrm{O}}_2^{\bar \cdot }$ production of cells at basal condition or stimulated with fMLP (10^−7^ M) was monitored kinetically (every 15 s) for 30 m.

Chemotaxis of neutrophils was performed using a 96-well modified Boyden chemotaxis chamber unit (Neuroprobe). Isolated PMNs were loaded with Calcein-Am, a cell-permanent dye, and incubated at 37 °C for 15 m. Cells were washed twice and resuspended at 3 × 10^6^/ml in 2% BSA in HBSS with Ca^2+^ and Mg^2+^ (Lonza). Chemoattractants, either fMLP (10^−7−10−10^ M), C5a (10^−8^ and 10^−10^ M),

IL-8 (10^−8−10−10^ M), or Substance P (10^−7^ and 10^−9^ M), were added to the bottom wells of the plate. The top filter unit was placed onto the plate and calcein-loaded PMNs (7.5 × 10^5^) were added on top of the filter. The plates were incubated at 37 °C for 45 m, and then the residual cells were aspirated from the top of the filter. PBS with EDTA (2 mM) was added to the top of the filter and then incubated at 4 °C for 30 m. The solution was aspirated from the top of the filter and then the plate was spun at 300 × g for 5 m to pellet the cells in the plate. The fluorescence in each well was determined and converted to the number of migrating cells based on a standard curve.

### RT-PCR

RNA was isolated from expanded T cell blasts, monocyte-derived macrophages, and THP-1 cells using the RNeasy Mini or RNeasy Plus Micro kits. Reverse transcription was performed using the iScript cDNA Synthesis Kit (Bio-Rad) using 1 μg of total RNA. RT-PCR was performed using the SsoAdvanced Universal SYBR Green Supermix (Bio-Rad) or iQ SYBR Green Supermix (Bio-Rad) using the Bio-Rad CFX96 Real-Time System.

### Mouse experiments

Mouse studies received ethical approval from Yale University’s Institutional Animal Care and Use Committee (IACUC) and were in compliance with all relevant ethical regulations for animal testing and research. Female and male 8- to 12- week-old wild-type C57BL/6 mice (Jackson Laboratories) were purchased. Female and male 8- to 12-week old *Pik3cg*−/− C57BL/6 mice were originally provided by Dr. Matthias Wymann and bred in-house. Female and male pet-store mice were purchased. Co-housing experiments occurred in a BSL-3 biocontainment facility within Yale University animal facilities. Pathogen testing was performed on fecal samples from pet-store and co-housed laboratory animals, testing for *Mycoplasma pulmonis*, Murine adenovirus K87, Murine adenovirus FL, Ectromelia virus, Pinworms (*Aspiculuris tetraptera* and *Syphacia obvelata*), *Pneumocystis murina, Streptobacillus moniliformis, Corynebacterium kutscheri, Encephalitozooan cuniculi*, Mouse parvovirus, Mouse hepatitis virus, Murine Astrovirus, Murine rotavirus EDIM, Pneumonia virus of mice, Reovirus 3, Mouse norovirus, Theiler’s murine encephalomyelitis virus, Sendai virus, Helicobacter spp., and *Spironucleus muris*. Prior to pet-store co-housing, WT and *Pik3cg*−/− mice were co-housed (females) or subjected to cage swaps (males) for 4 weeks. Cage swaps were performed by placing WT mice into the *Pik3cg*−/− mice cage and *Pik3cg*−/− mice into the WT mice cage, with transfers being made daily. After 4 weeks, pet-store mice of the same sex were introduced to the cages (female) or incorporated into daily cage swaps (male) for 56 days. Laboratory mice were weighed daily for 14 days, and then weekly for the next 6 weeks.

For flow cytometry, mouse blood was collected in Microtainer tubes with EDTA and then transferred to a 96-V bottom well plate. Cells were surface stained with PerCP/Cyanine 5.5-conjugated anti-CD183 (BioLegend), PE/Cy7-conjugated anti-CD8α (BioLegend), PE-conjugated anti-Klrg1 (BioLegend), APC/Fire 750-conjugated anti-CD44 (BioLegend), Alexa Fluor 647-conjugated anti-CD62L (BioLegend), Brilliant Violet 650-conjugated anti-CD19 (BioLegend), Pacific Blue-conjugated anti-CD4 (BioLegend), APC-conjugated anti-CD25 (BioLegend), PerCP/Cyanine 5.5-conjugated anti-CD4 (BioLegend), PE-conjugated Foxp3 (BioLegend), FITC-conjugated IL-10 (BioLegend), and Brilliant Violet 650-conjugated anti-IFNγ (BioLegend).

For mouse immunoglobulin analysis, blood was collected in Microtainer tubes without any additives and allowed to clot at RT for 30 min. Tubes were centrifuged at 1000 × g for 10 min and serum was transferred to new Eppendorf tubes and stored at −80 °C. Serum samples were analyzed using the bead-based immunoassay Mouse Immunoglobulin Isotyping LEGENDPlex (BioLegend). The selected panel of capture beads used were IgG1, IgG2a, IgG2b, IgG3, IgA, and IgM, and manufacturer instructions were followed. Serum samples were also analyzed using the Mouse Inflammation Panel LEGENDplex (BioLegend). The selected panel of capture beads used were GM-CSF, IFN-β, IL-17A, IL-27, IL-6, IL-10, IL-1β, IL-12p70, MCP-1, TNF-α, IFNγ, IL-1α, and IL-23, and manufacturer instructions were followed.

For mouse T cell quantification in the small intestine and lung, mice were sacrificed at day 56 and dissected in which small intestine and lung were separated and suspended in 10% Formalin solution (Sigma-Aldrich). Samples were sent to Histowiz for immunohistochemistry (IHC) staining of CD3+ cells in the tissue. For small intestine analysis, ten random 200 μm-sized images of sections for each mouse were randomly assigned a number. For lung analysis, twenty random 100 μm-sized images of sections for each mouse were randomly assigned a number. All images were hand-counted and quantified. After collection, the count for CD3+ cells in each image was assigned to the correct mouse sample, and the ten (small intestine) or twenty (lung) images for each mouse were averaged to get total CD3 + T cells per section in each mouse.

For mouse T-Reg quantification, mice were sacrificed at day 56 and the spleen was separated and mashed over a 70 micron filter in a suspension of cRPMI. Cells were washed with PBS, and then suspended in ACK lysis buffer (Quality Biological/VWR). Following red-blood cell lysis, cells were washed with media and then resuspended and counted. Approximately 16 million cells from each mouse were activated with 1 μg/mL PMA (Caymen Chemical), 1 μg/mL Ionomycin (Caymen Chemcal), and 5 μg/mL Brefeldin A (Sigma-Aldrich). After incubation for 6 h, cells were spun down and resuspended in FACs buffer and 4 million cells each were stained for T_reg_ cells panels.

### Statistical tests

The number of samples per group and experiment repeats, as well as the statistical tests used is indicated in each figure legend.

### Reporting summary

Further information on research design is available in the Nature Research Reporting Summary linked to this article.

## Supplementary information

Supplementary info

Reporting Summary

## Data Availability

Whole-exome sequencing data are available through dbGaP accession number phs001848.v1.p1, and mutations will be automatically archived by Online Mendelian inheritance in Man (OMIM) once the paper is published.
