# Supplementary material for: Human PI3Kγ deficiency and its microbiota-dependent mouse model reveal immunodeficiency and tissue immunopathology
Source: Nat Commun. 2019 Sep 25;10:4364. doi: 10.1038/s41467-019-12311-5 (PMC6761123; doi:10.1038/s41467-019-12311-5)
Supplement: Supplementary file 1 — Supplementary info [file 41467_2019_12311_MOESM1_ESM.pdf]

Human PI3Kγ deficiency and its microbiota-dependent mouse model reveal immunodeficiency and tissue immunopathology

Short title: PI3Kγ deficiency with immune dysregulation

Andrew J. Takeda[1,#], Timothy J. Maher[1,#], Yu Zhang[2,3], Stephen M. Lanahan[1], Molly L. Bucklin[1], Susan R. Compton[4], Paul M. Tyler[1], William A. Comrie[2], Makoto Matsuda[5], Kenneth N. Olivier[6], Stefania Pittaluga[7], Joshua J. McElwee[8], Debra A. Long Priel[9], Douglas B. Kuhns[9], Roger L. Williams[5], Peter J. Mustillo[10], Matthias P. Wymann[11], V. Koneti Rao[3], and Carrie L. Lucas[1]*

[#]Equal contributors

*Corresponding author: Carrie.Lucas@yale.edu; 203-785-7158

Supplementary Information

SUPPLEMENTARY FIGURES

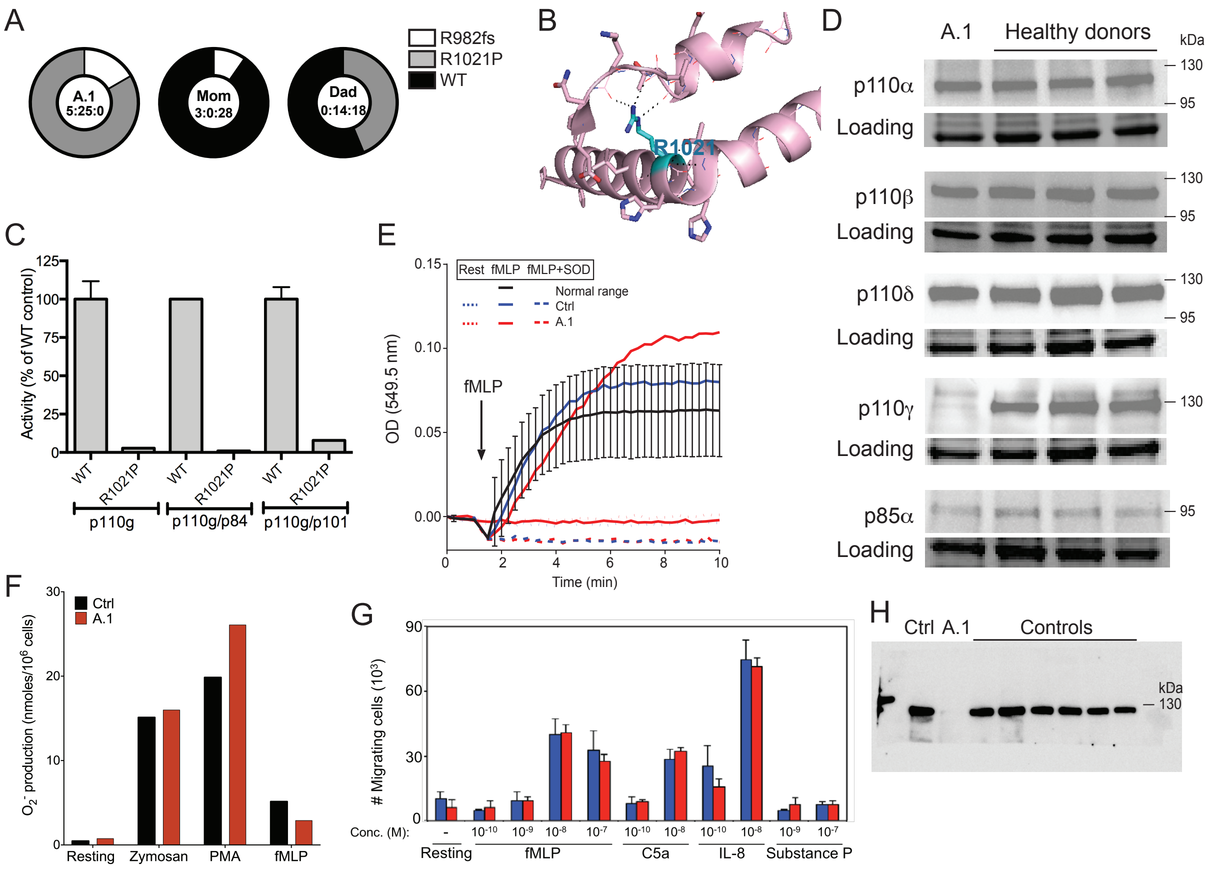

Supplementary Figure 1. Detailed characterization of inherited *PIK3CG* alleles.

A, Relative frequency of mRNA from each *PIK3CG* allele in T cell blasts from the indicated subject determined by Sanger sequencing of individual clones. **B**, Hydrogen bonds formed by the R1021 residue in the kinase domain of wild-type p110γ protein depicted in a ribbon diagram. **C**, *In vitro* lipid kinase activity of recombinant p110γ wild-type protein or R1021P variant encoded by paternally inherited *PIK3CG* allele alone or in complex with the indicated p84 or p101 regulatory subunit. **D**, Representative western blots for p110α, p110β, p110δ, p110γ, and p85α in T cell blasts from three unrelated healthy control and patient A.1. Equal protein loading was assessed using stain-free imaging (Bio-Rad). Data are representative of three independent experiments for p110γ and representative of two independent experiments for the others. **E, F**, Cytochrome c reduction and superoxide production by neutrophils from unrelated healthy control or patient A.1 without stimulation (Resting) or after stimulation with the indicated stimuli. **G**, Chemotaxis of neutrophils toward the indicated stimuli in a modified Boyden chamber. Data in E, F, and G are representative of three independent experiments. **H**, Uncropped image of blot for p110γ in patient A.1 compared to controls (independent experiment as in Figure 1E and Supplementary Figure 1D).

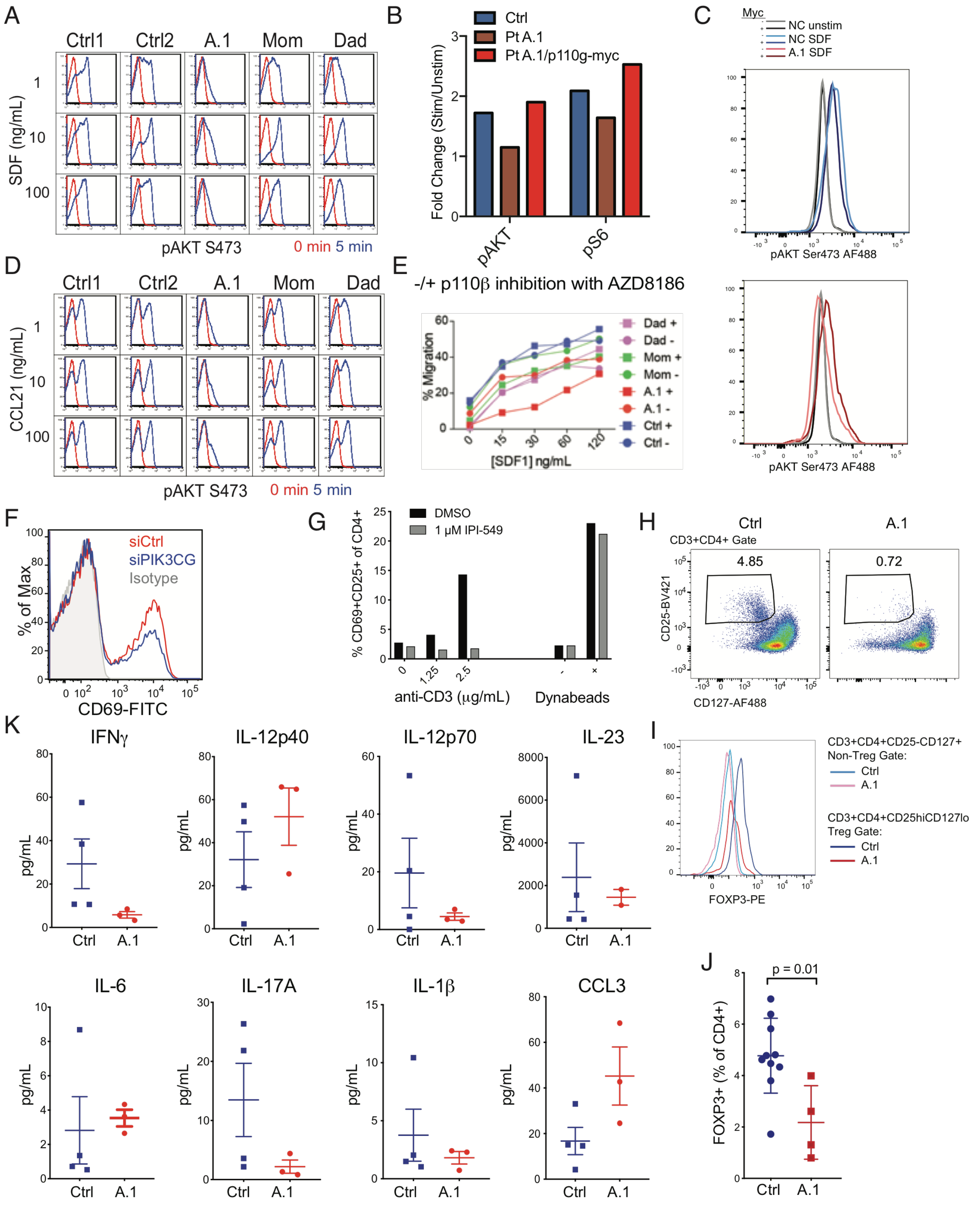

Supplementary Figure 2. Extended T cell and serum analyses in patient A. 1.
A, Phosphorylation of AKT Ser473 in expanded T cell blasts/effectors stimulated with indicated concentrations of SDF. Data are representative of three independent experiments. **B**, AKT and S6 phosphorylation in untransfected T cells from control subject or patient, or patient T cells

transfected with p110γ-Myc expression vector. Data are presented as mean of the fold change of SDF-stimulated to unstimulated cells and are representative of two independent experiments (n = 1 for each group). **C**, Representative flow cytometry histograms of SDF-stimulated AKT phosphorylation in untransfected T cells from control subject or patient, or patient T cells transfected with p110γ-Myc expression vector. Data are representative of two independent experiments. **D**, CCL21-stimulated AKT phosphorylation in T cell blasts as in A. Data are representative of three independent experiments. **E**, Transwell chemotaxis in response to an SDF-1 concentration gradient with 50 μM AZD8186 to inhibit p110β. **F**, Representative flow cytometry histogram of CD69+ T cells from a healthy control subject stimulated as for 24 hr with anti-CD3 and anti-CD28 after transfection with siRNA targeting *PIK3CG* mRNA or non-specific siRNA. E and F are representative of two independent experiments. **G**, Frequency of CD69+CD25+ of CD4+ T cells treated with IPI-549 (1 μM) or DMSO control as indicated and activated with indicated concentrations of anti-CD3 and anti-CD28 (2 μg/mL) or PBMCs treated in similar manner as T cells and activated with dynabeads (high signal strength). Data are presented as mean of individual cell isolates and are representative of two independent experiments (n = 1 for each group). **H-I**, Flow cytometric analysis of Treg cells showing surface staining for CD25 and CD127 among CD3+CD4+ cells and intracellular FOXP3 staining among the non-Treg and Treg gate for a representative healthy control donor and patient A.1. **J**, Frequency of FOXP3+ cells among peripheral blood CD4+ T cells in unrelated healthy controls (n = 10) and patient A.1 (n = 4). Data from four independent experiments are presented as mean ± SD. Statistical analysis was performed using two-tailed unpaired T-test. **K**, Concentrations of the indicated cytokine or chemokine in serum from independent blood draws of unrelated healthy controls (n = 4) and patient A.1 (n = 3). Data from three independent experiments are presented as mean ± SEM.

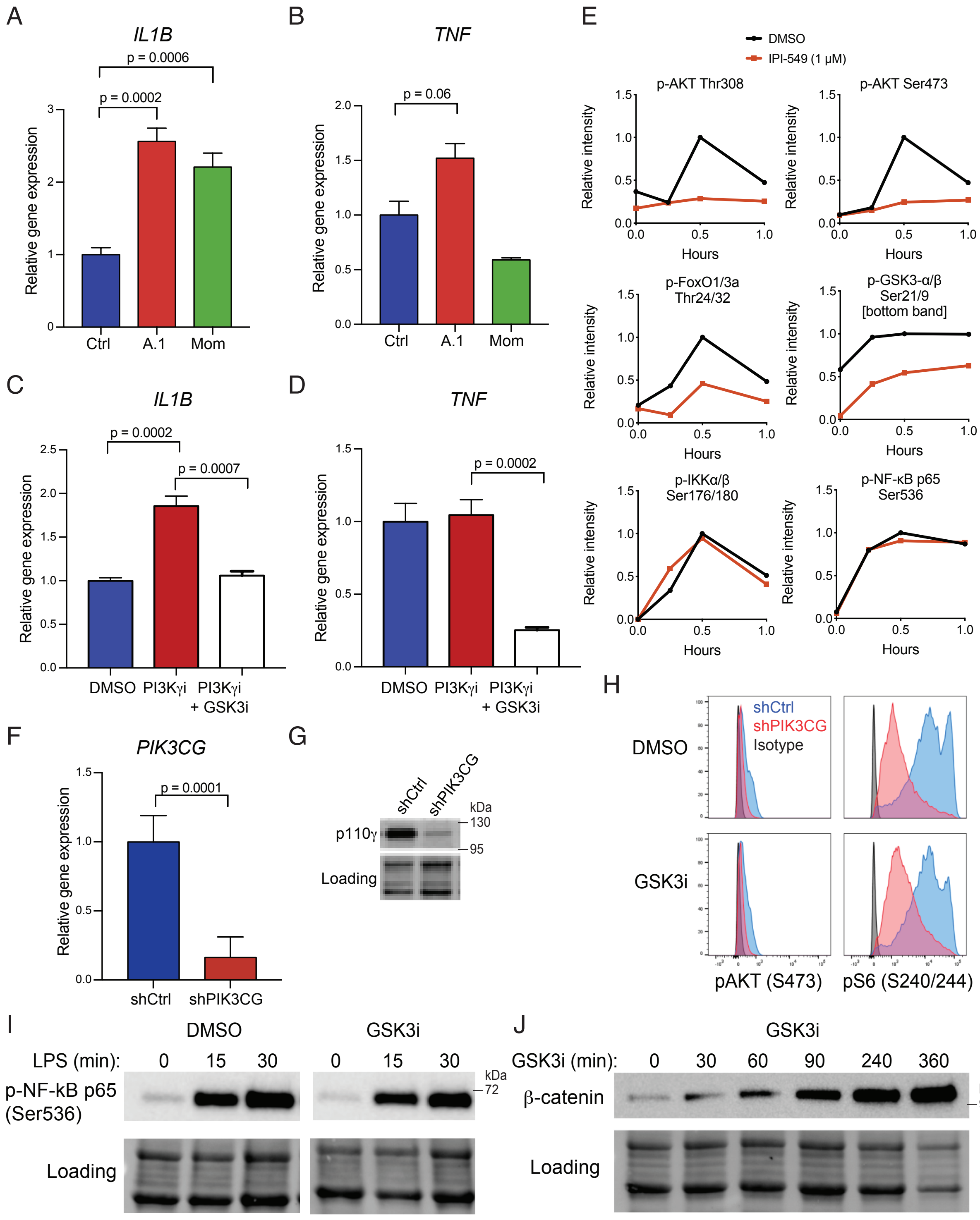

Supplementary Figure 3. Detailed analysis of myeloid cells with PI3Kγ deficiency, inhibition, or knockdown.

A-B, Gene expression normalized to *GAPDH* from monocyte-derived macrophages stimulated with LPS and IFNγ as in Fig. 3A. Data are presented as mean ± SD and are representative of two independent experiments, in which one primary cell isolate for each group was run with three technical replicates. Statistical analysis was performed using a two-tailed unpaired T-test.

C-D, Gene expression normalized to *GAPDH* from PMA-differentiated THP-1 macrophages treated as indicated and stimulated with LPS and IFNγ as in Fig. 3E. Data are presented as mean ± SD and are representative of three (C) or two (D) independent experiments, in which one primary cell isolate for each group was run with three technical replicates. Statistical analysis was performed using two-tailed unpaired T-test. **E,** Relative intensity from western blots of pAKT (T308), pAKT (S473), pFoxo1/3a, pGSK3α/β, pIKKα/β, pNF-kB p65 in THP1 monocytes treated with PI3Kγi IPI-549 (1 μM) and stimulated with LPS (100 ng/mL). **F**, mRNA expression of *IL12B* normalized to *GAPDH* and *RPL37A* in in THP-1 macrophages expressing *PIK3CG*-targeted or control shRNA treated as in E and stimulated for 8 hr with IFNγ (20 ng/mL) and LPS (100 ng/mL). Data are presented as mean ± CI and are representative of two independent experiments, in which one primary cell isolate for each group was run with three technical replicates. Statistical analysis was performed using two-tailed unpaired T-test. **G**, Representative western blot of protein expression of p110γ in THP-1 macrophages stably expressing *PIK3CG*-targeted or control shRNA. Data are representative of two independent experiments. **H,** Flow cytometry histograms of pAKT and pS6 in THP-1 macrophages expressing *PIK3CG*-targeted or control shRNA treated with GSK3i LY2090314 (20 nM) or DMSO control. Data are from one experiment. **I, J**, Representative western blots of indicated proteins from THP-1 monocytes stimulated with LPS (100 ng/mL) and treated with GSK3i LY2090314 (20 nM) or DMSO control. Data are from one experiment (I) or representative of five experiments (J). For G, I, and J, equal protein loading was assessed using stain-free imaging (Bio-Rad).

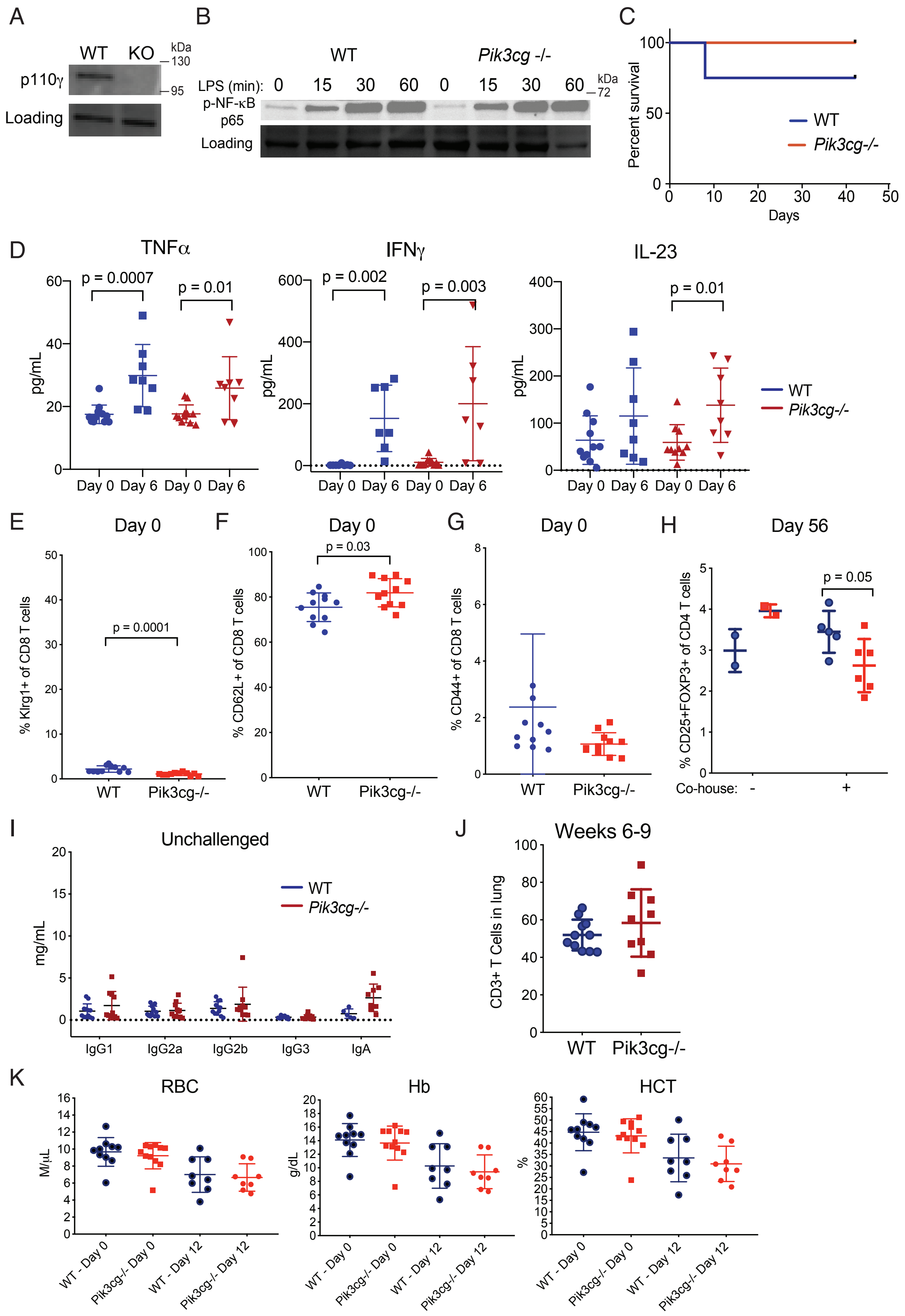
A
WT
KO
kDa
130
95
p110γ
Loading
B
WT
Pik3cg -/-
LPS (min): 0 15 30 60 0 15 30 60
kDa
72
p-NF-κB p65
Loading
C
Percent survival
100
50
0
0 10 20 30 40 50
Days
WT
Pik3cg-/-
D
TNFα
pg/mL
p = 0.0007
p = 0.01
Day 0 Day 6 Day 0 Day 6
IFNγ
p = 0.002
p = 0.003
IL-23
p = 0.01
WT
Pik3cg-/-
E
Day 0
% Klrg1+ of CD8 T cells
p = 0.0001
WT
Pik3cg-/-
F
Day 0
% CD62L+ of CD8 T cells
p = 0.03
G
Day 0
% CD44+ of CD8 T cells
H
Day 56
% CD25+FOXP3+ of CD4 T cells
p = 0.05
Co-house: - +
I
Unchallenged
mg/mL
WT
Pik3cg-/-
IgG1 IgG2a IgG2b IgG3 IgA
J
Weeks 6-9
CD3+ T Cells in lung
WT
Pik3cg-/-
K
RBC
M/μL
Hb
g/dL
HCT
%
WT - Day 0
Pik3cg-/- Day 0
WT - Day 12
Pik3cg-/- Day 12

Supplementary Figure 4. *Pik3cg* KO mouse analyses.

A, p110γ protein expression by wild-type and *Pik3cg*-/- mouse bone marrow-derived macrophages. Data are from one experiment. **B**, Representative western blot of two independent experiments of NF-κB p65 from WT and *Pik3cg*-/- murine bone-marrow derived macrophages stimulated with LPS (100 ng/mL) for indicated time. For A and B, equal protein loading was assessed using stain-free imaging (Bio-Rad). **C**, Kaplan-Meier survival analysis of mice co-housed with pet-store mice. Data are representative of four independent experiments. **D**, Concentration of indicated cytokines in the serum of co-housed mice from day 0 and day 6. Data are presented as mean ± SD and are representative of four independent experiments (n = 8-12 for day 0, n = 7-8 for day 6) Statistical analysis was performed using two-tailed unpaired T-test. **E-G**, Frequency of Klrg1+, CD62L+, and CD44+ among CD8+ T cells from mice at day 0. Data are presented as mean ± SD and are representative of four independent experiments (n = 11 for each group). Statistical analysis was performed using two-tailed unpaired T-test. **H**, Frequency of CD25+FOXP3+ cells among CD4+ T cells after PMA/ionomycin stimulation of splenocytes from WT (n = 2 for non-co-housed and n = 5 for co-housed) and *Pik3cg*-/- (n = 2 for non-co-housed and n = 6 for co-housed) after 56 days. Data are presented as mean ± SD. Statistical analysis was performed using two-tailed unpaired T-test. **I**, Concentration of antibodies in serum from unchallenged mice at weeks 3 to 5. Data are presented as mean ± SD and are summarized from four independent experiments (n = 5-12 for each group). **J**, Total number of CD3+ T cells per 100 μm section of immunohistochemistry staining of the lung from WT vs. *Pik3cg*-/- mice after 56 days of exposure to pet-store mice. Data are presented as mean ± SD and are cumulative data from two independent experiments (n = 11 for WT, n = 9 for *Pik3cg*-/-). **K**, Complete blood count analysis on the indicated animals with 0 or 12 days of exposure to pet-store mice, depicting red blood cell (RBC) count, hemoglobin (Hb), and hematocrit (HCT) (n = 8-10 for WT, n = 8-11 for *Pik3cg*-/-).

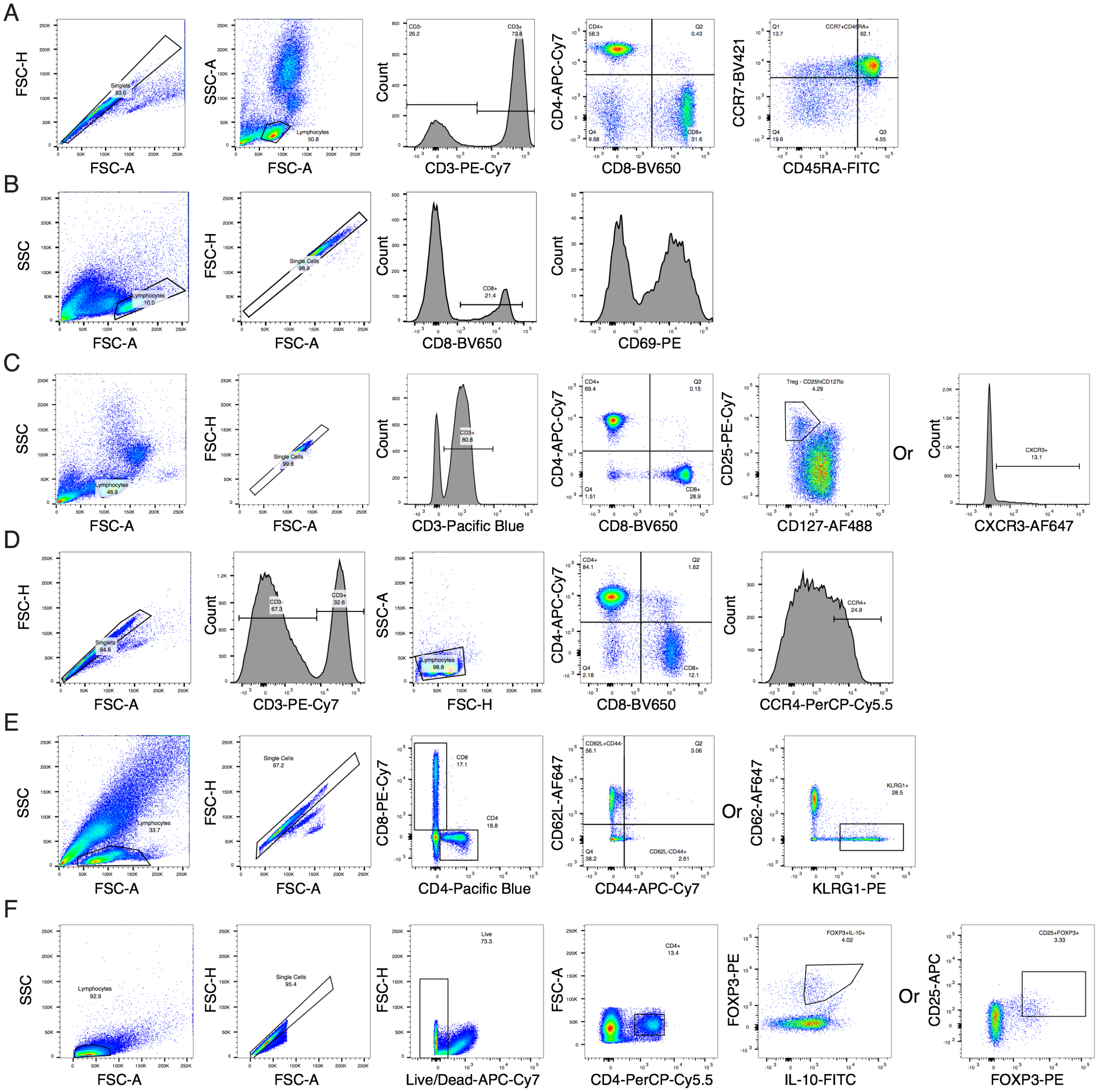

Supplementary Figure 5. Supplemental Gating Strategies.
A, Gating strategy for T cells staining for CD4, CD8, CD45RA, and CCR7 as seen in Figure 2A. **B**, Gating strategy for CD69 expression on CD8+ T cells for Figure 2B. **C**, Gating strategy for CD4+ peripheral blood T cells frequency of CXCR3+ or CD25^{hi}CD127^{lo} for figure 2C and 2E. **D**, Gating strategy for CD4+ peripheral blood T cells frequency of CCR4+ for figure 2D. **E**, Gating strategy for CD8+ peripheral blood T cells frequency of CD62L+, CD44+, or Klrg1+ for figures 4D, 4E, 4F, S4E, S4F, S4G. **F**, Gating strategy for CD4+ T cells frequency of FOXP3+IL-10+ or CD25+ for figures 4G and S4H.

**Mouse pathogen**	**Pet-store mice**	**Laboratory mice after co-housing**
Mouse parvovirus	+	-
Mouse hepatitis virus	+	+
Theiler’s murine encephalomyelitis virus	+	-
Murine Astrovirus	-/+	-/+
Murine Norovirus	-/+	-/+
Helicobacter spp.	+	+
Mycoplasma pulmonis	-	-
Murine adenovirus K87	-	-
Pinworms (*Aspiculuris tetraptera* and *Syphacia obvelata*)	-/+	-/+
Ectromelia virus	-	-
Pneumocystis murina	-	-
Streptobacillus moniliformis	-	-
Corynebacterium kutscheri	-	-
Encephalitozooan cuniculi	-	-

Supplementary Table 1. Pathogen testing results in pet-store and co-housed laboratory mice.

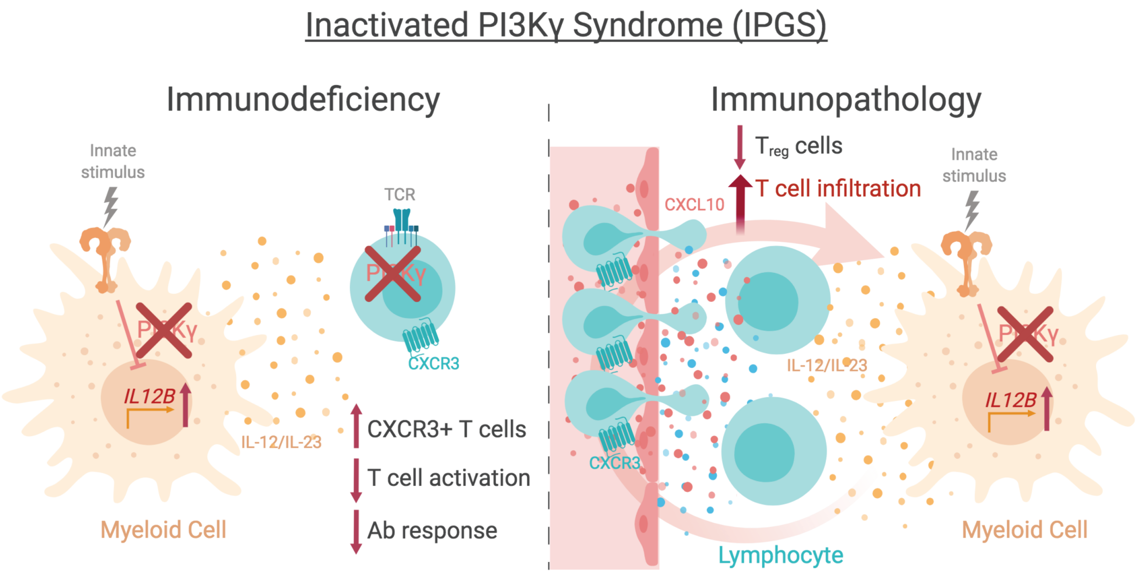

Supplementary Figure 6. Schematic model of mechanisms driving immunodeficiency and immunopathology from PI3Kγ deficiency.
